# Virtual reality for the management of musculoskeletal pain: an umbrella review

**DOI:** 10.3389/fmed.2025.1572464

**Published:** 2025-07-09

**Authors:** Sultan Kalikanov, Aliya Baizhanova, Meiram Tungushpayev, Dmitriy Viderman

**Affiliations:** ^1^Department of Education, “University Medical Center” Corporate Fund, Astana, Kazakhstan; ^2^Department of Surgery, School of Medicine, Nazarbayev University, Astana, Kazakhstan; ^3^Department of Anesthesiology, Intensive Care and Pain Medicine, National Research Oncology Center, Astana, Kazakhstan

**Keywords:** virtual reality, pain, musculoskeletal pain, low back pain, neck pain

## Abstract

**Background:**

Musculoskeletal pain (MSK) is a condition that affects multiple parts of the musculoskeletal system, including limbs, neck, and back, leading to deterioration in both mental and physical health and overall quality of life. Despite the available treatments, they are not considered effective enough to eradicate pain symptoms, thereby requiring new methods as a substitute. This review comprehensively summarizes virtual reality (VR) technology as an adjunct or an alternative treatment for MSK pain and aims to explore the most suitable conditions and settings of VR.

**Methods:**

Pubmed, Scopus, and Cochrane databases were searched for recent systematic reviews and meta-analyses investigating VR and MSK pain. The search was performed according to PRISMA (Preferred Reporting Items for Systematic Reviews and Meta-Analyses) guidelines and revealed 17 relevant articles. The AMSTAR-2 (A MeaSurement Tool to Assess systematic Reviews) analysis was conducted to assess the quality of included studies. The Corrected Covered Area was calculated to identify the degree of overlap.

**Results:**

The results found significant pain reduction and mental and physical improvements in patients with MSK pain in comparison to standard therapies in treating neck, knee, and back pain. Nevertheless, the heterogeneity and inconsistencies in results among papers were recognized. The promising aspects are multimodality, namely, VR in combination with exercises, patient acceptance of VR, and the effectiveness of immersive, non-immersive, and gamified versions. These findings also revealed the need for more research on underexplored regions, standardized methodologies, and personalized approaches.

**Conclusion:**

To summarize, VR poses the potential to treat MSK pain as an adjunct, and future research is recommended to focus on improving methodological rigor and multimodal approaches.

**Systematic review registration:**

OSF (https://osf.io/uyc7z).

## 1 Introduction

Chronic musculoskeletal pain (CMP) is a pervasive condition characterized by persistent gnawing muscle discomfort sensations, affecting around 20–30% of the general population (1). Such pain severely limits mobility and interferes with daily activities, adversely affecting the quality of life. Musculoskeletal (MSK) pain is of multifactorial origin and is commonly associated with the neck, shoulder, hips, lower back, and knee regions (2). Many individuals end up discontinuing their treatments following a prolonged treatment period despite the numerous treatment options that are available for such pain, suggesting that the current treatment approaches are inadequate to deal with this condition (3).

Virtual reality (VR) is a simulation that is generated by a computer and allows interaction with immersive and realistic environments through purpose-built hardware and software (4). VR creates a sense of presence inside the environments that are usually impractical or inaccessible in real life. Deep immersion into a virtual environment can effectively divert attention from painful stimuli, promoting analgesic and anxiolytic effects (4). The therapeutic potential of VR has already been demonstrated in various settings, including acute pain management, burn injuries, wound care, chemotherapy, physical therapies, and mental health disorders (5–9). This therapeutic effect is primarily achieved by providing a contrasting environment to the injury setting, such as presenting burn injury patients with cold landscapes through VR (5). By altering the contextual setting of the pain source, VR can effectively downregulate pain signals, reducing conscious sensations of pain (10). The ability to gamify virtual reality could increase satisfaction and motivation to continue treatment, improving treatment prospects (10).

Virtual reality demonstrates significant promise as an adjunct to conservative therapies, garnering substantial interest in pain management settings. The evidence advocates for using non-immersive, immersive, or mixed types of VR, each differing in immersion intensity (11). A non-immersive VR allows interaction using a mouse or hand-held device to interact with a computer-generated reality of oneself while still seeing and interacting with the outside world (12). Immersive VR, on the other hand, may be composed of multiple pieces of equipment, such as a stereoscopic headset or haptic device, requiring whole-body movement and cognitive effort (11). In cases where conventional therapies are insufficient or a need to switch to a more aggressive treatment arises, VR might serve as a new perspective on pain management tools.

The use of VR in managing musculoskeletal pain is a relatively new area and requires substantial research. Most studies on its application are inconclusive and are typically constrained by the heterogeneity of VR types. Moreover, the question of which VR conditioning and setting would be most advantageous for patients with different anatomical MSK pain regions remains crucial. This umbrella review aims to evaluate the effectiveness of VR in reducing musculoskeletal pain and related secondary outcomes across different body regions, compared to conventional treatments, and to identify VR key parameters such as immersion and gamification.

## 2 Methods

The protocol of the study was submitted and registered in the Open Science Framework registry (registration doi: https://doi.org/10.17605/OSF.IO/GAJ84). The umbrella review followed the PRISMA statement (13).

### 2.1 Search strategy

We conducted a systematic search in Scopus, PubMed, and Cochrane Database of Systematic Reviews databases from their inception until September 5, 2024, to locate systematic reviews and meta-analyses examining the effects of virtual reality on MSK pain management. We manually reviewed the citations in the selected eligible papers to identify any additional relevant studies that might have been missed in the initial search. We followed the PRISMA (Preferred Reporting Items for Systematic Reviews and Meta-Analyses) guidelines for this umbrella review. The search terms used included: “virtual reality,” “virtual,” “reality,” “pain,” and “systematic review.”

The full search strings were:

PubMed:

(“virtual reality”[Title/Abstract] OR “virtual”[Title/Abstract] OR “reality”[Title/Abstract]) AND (“pain”[Title/Abstract]) AND (“systematic review”[Publication Type] OR “systematic review”[Title/Abstract])

Cochrane library:

(“virtual reality” OR “virtual” OR “reality”) AND (“pain”) AND (“systematic review”)

Scopus:

TITLE-ABS-KEY(“virtual reality” OR “virtual” OR “reality”) AND TITLE-ABS-KEY(“pain”) AND TITLE-ABS-KEY(“systematic review”)

### 2.2 Inclusion and exclusion criteria

We followed the PICO criteria:

- Patients: patients with MSK pain.
- Intervention: virtual reality.
- Control: placebo or traditional treatment.
- Outcome: efficacy of pain management with secondary outcomes including disability and kinesiophobia.

The inclusion criteria were:

- Study types: systematic reviews and meta-analyses.
- Research focus: VR for MSK pain management.
- Publications in peer-reviewed journals.
- Language: English.

Exclusion criteria included:

- Study designs: observational studies, randomized controlled trials, animal studies, editorials, and correspondence.

### 2.3 Literature screening and data extraction

Two authors independently conducted literature screening based on inclusion and exclusion criteria. Firstly, abstracts and titles were read. Next, full manuscripts were retrieved for further evaluation. Any disagreements were resolved through discussion. For data extraction and analysis, both authors independently recorded the following information of included studies in a table:

- Author and citation.
- Study design.
- Types of MSK pain.
- Short description of the protocol.
- Number of patients in each meta-analysis.
- Total number of studies in each meta-analysis.
- Reported benefits of VR in MSK pain.
- Complications.
- Mechanism of VR.
- Study conclusions.

### 2.4 Methodological quality assessment of the systematic reviews

The updated second version of AMSTAR-2 (A MeaSurement Tool to Assess systematic Reviews) was used to assess the methodological quality of systematic reviews (14). The AMSTAR-2 was performed by two authors independently and compared with each other. Any discrepancies were resolved through discussion. Each item was rated as “Yes” (meets the standard), “No” (does not meet the standard), “Partial yes” (meets the standard with some limitations), and “Not applicable” (e.g., no meta-analysis conducted). The overall confidence in the quality of each review was classified as high when there was no or only one non-critical flaw; moderate when there was more than one non-critical flaw; low when there was one critical flaw with or without non-critical flaws; and critically low when there was more than one critical flaw. This classification structure was applied consistently to all included reviews. The critical domains are items 2, 4, 7, 9, 11, 13, 15, and non-critical domains are items 1, 3, 5, 6, 8, 10, 12, 14, 16, which are stated in Table 1.

**Table 1:** AMSTAR-2 analysis of included studies.

Author, citation	1	2	3	4	5	6	7	8	9	10	11	12	13	14	15	16
Ye et al., Canada, (20)	+	Partial Yes	−	Partial Yes	−	+	−	Partial Yes	+	−	+	+ (low)	+	+ (discussed)	−	+
Mo et al., China, (21)	+	Partial Yes	−	Partial Yes	+	+	−	+	+	−	+	- (high)	+ (high)	+ (I^2^ = 0%)	− (small sample size to generate funnel plots)	+
Guo et al., China, (19)	+	+	−	Partial Yes	−	+	−	+	+	−	+	+ (subgroup analyses)	+	+	−	+
Choi et al., Korea, (18)	+	+	+	+	+	+	+	Partial Yes	+	−	+	+ (low)	+	+ (impact)	+	+
Kantha et al., Taiwan, (23)	+	+	−	Partial Yes	+	+	−	+	+	−	+	+ (good quality)	+	+ (I^2^ = 0%)	−	+
Gava et al., Brazil, (24)	+	+	−	Partial Yes	+	+	−	+	+	−	+	+ (subgroup analyses)	+ (quality of evidence)	+ (source)	− (small sample size to generate funnel plots)	+
Brea-Gómez et al., Spain, (17)	+	+	−	Partial Yes	+	+	−	+	+	−	+	+	+	+	−	+
Bordeleau et al., Canada, (16)	+	+	−	Partial Yes	+	+	+	+	+	+	+	+	+ (high, discussed)	+ (I^2^ = 85%, discussed)	+	+
Collado-Mateo et al., Spain, (22)	+	+	−	Partial Yes	+	+	−	+	+	−	+	+ (low quality, assessed)	+	+ (I^2^ = 82%, discussed)	−	+
Lo et al., China, (25)	−	+	−	Partial Yes	+	+	+	+	+	−	+	+	−	+	− (small sample size to generate funnel plots)	+
Henríquez-Jurado et al., Spain, (26)	+	+	−	+	+	+	−	Partial yes	+	+	+	+	+	+	+	+
Li et al., China, (27)	−	Partial yes	Partial yes	+	+	+	−	+	+	−	+	+	+	+	Partial yes	+
Wong et al., China, (28)	+	Partial yes	Partial yes	Partial yes	−	−	−	+	+	+	No meta-analysis conducted	No meta-analysis conducted	−	−	No meta-analysis conducted	+
Guo et al., China, (29)	−	+	Partial yes	+	+	−	−	+	+	−	+	+	−	−	+	+
Hao et al., USA, (30)	+	Partial yes	Partial yes	+	+	+	−	+	+	+	+	+	−	−	−	+
Zhang et al., China, (31)	+	Partial yes	Partial yes	+	+	+	+	+	+	+	+	+	−	+	−	+
Opara and Kozinc, Slovenia, (32)	+	+	Partial yes	+	+	+	−	+	+	+	+	+	+	+	−	+

MA, Meta-analysis; SR, Systematic review; RoB, Risk of bias.

1. Did the research questions and inclusion criteria for the review include the components of PICO?

2. Did the report of the review contain an explicit statement that the review methods were established prior to the conduct of the review and did the report justify any significant deviations from the protocol?

3. Did the review authors explain their selection of the study designs for inclusion in the review?

4. Did the review authors use a comprehensive literature search strategy?

5. Did the review authors perform study selection in duplicate?

6. Did the review authors perform data extraction in duplicate?

7. Did the review authors provide a list of excluded studies and justify the exclusions?

8. Did the review authors describe the included studies in adequate detail?

9. Did the review authors use a satisfactory technique for assessing the risk of bias (RoB) in individual studies that were included in the review?

10. Did the review authors report on the sources of funding for the studies included in the review?

11. If meta-analysis was performed did the review authors use appropriate methods for statistical combination of results?

12. If meta-analysis was performed, did the review authors assess the potential impact of RoB in individual studies on the results of the meta-analysis or other evidence synthesis?

13. Did the review authors account for RoB in individual studies when interpreting/discussing the results of the review?

14. Did the review authors provide a satisfactory explanation for, and discussion of, any heterogeneity observed in the results of the review?

15. If they performed quantitative synthesis did the review authors carry out adequate investigation of publication bias (small study bias) and discuss its likely impact on the results of the review?

16. Did the review authors report any potential sources of conflict of interest, including any funding they received for conducting the review?

### 2.5 Overlap assessment

The Corrected Covered Area (CCA) was calculated to identify the degree to which the same primary studies had been included across systematic reviews and meta-analyses. The formula is described by Kirvalidze et al. (15):


$$\mathrm{CCA}=(N-r)/(r^{*}c)-r$$

where N stands for the total number of primary studies that appeared, including double counting, r is the number of unique primary studies, and c is the number of systematic reviews that are included in the umbrella review. Consequently, the citation matrix was built to provide measurements for CCA. The classification of overlap degree is as follows: 0–5% is slight overlap, 6–10% is moderate overlap, 11–15% is high overlap, and more than 15% is very high overlap (15).

### 2.6 Data synthesis

The summarization and comparison of the findings from the included studies were performed via a narrative synthesis. The outcomes were grouped thematically based on pain localization, VR immersion, gamification, and mechanisms. The overall synthesis was supported by structured tables to summarize quantitative findings and methodological quality, as well as by figures to visually condense the key results.

## 3 Results

### 3.1 Study selection and patient characteristics

**Figure 1:**
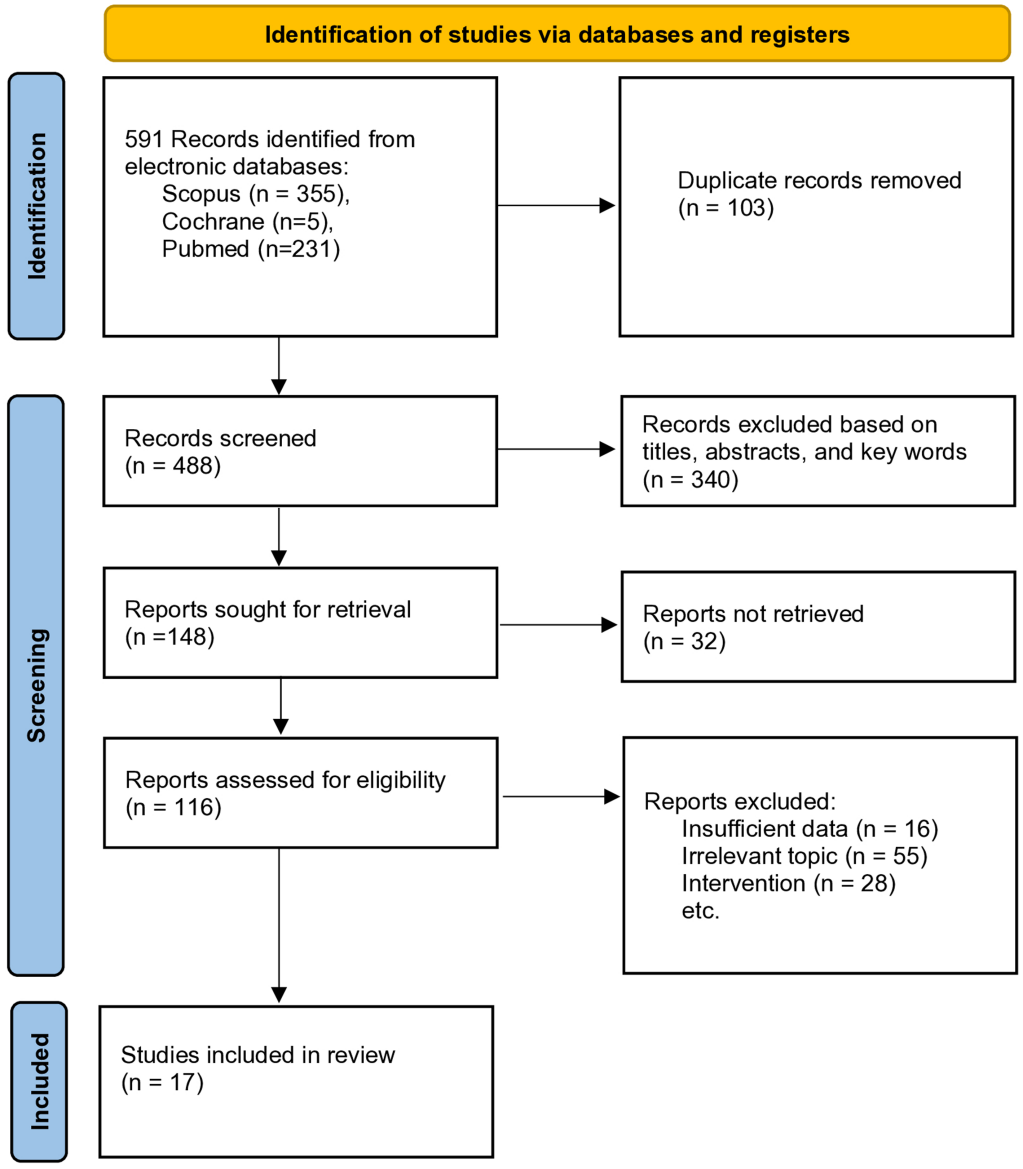
PRISMA flow diagram.

In total, 591 articles were found in 3 databases. A total of 103 duplicates were identified and removed manually. After abstract screening, 340 articles were retained and 99 articles were excluded due to the following reasons: (1) the information was incomplete, (2) the focus of the article was unrelated to the subject of interest, (3) and the intervention did not use VR therapy. This review selected in total 17 studies (16–32) published between 2017 and 2024, with a cumulative sample of 11,638 participants. The PRISMA flowchart of selecting the articles for inclusion is depicted in Figure 1. Four articles included participants suffering from chronic lower back pain (16–18, 27), four articles with neck pain (19, 20, 30, 32) and two articles included patients with both lower back and neck pain (26, 28). One article focused on chronic spinal pain and inflammation (30), and one investigated knee joint pain (29). Two articles (23, 25) focused on immersion, and three articles (21, 22, 24) on exergames to treat MSK pain. Only one article conducted a systematic review without meta-analysis (28), while the other 16 studies conducted both a systematic review and meta-analysis. Most studies primarily assessed pain intensity using Visual Analog Scale (VAS) and Numeric Rating Scale (NRS). Less commonly reported pain measures were Defense and Veterans Pain Rating Scale (DVPRS) and Pain Pressure Threshold (PPT). The detailed characteristics of the included studies (study groups, sample size, protocol description, reported benefits, complications, mechanisms, and conclusions) are summarized in the Supplementary Table 1.

### 3.2 Characteristics of VR regimen, equipment types, and settings, and methodological quality

The immersion intensity and duration of VR therapy varied greatly between studies. Four studies utilized both immersive and non-immersive VR (23, 25, 26, 28). The types of equipment that were mostly reported to be used in experimental VR groups included mobile phones with VR apps (16, 31), game consoles (25, 27, 28), Nintendo Wii motion tracks (16, 17, 20, 22–25, 27, 31), simulators (16, 22, 25, 27), headsets (18, 20), balance boards simulators (16, 22), television with motion sensors (25), and playing glasses (16, 17, 24). Therapies such as kinematic training, stretching, and standard rehabilitation were mainly used as controls. The duration of VR therapy ranged between 6 min to 90 min, with participants having between 1 to 7 training sessions per week. Notably, approximately 30 min’ duration was found to be average, and one article (28) proposed 20 min as the threshold for the discomfort of using VR. The total treatment length comprised the shortest of 3 days (16) in back pain rehabilitation and the longest of 24 weeks (23, 25, 27, 28, 31, 32). These lengths were utilized to specifically analyze the immediate (days) and long-term effects (half a year), whereas the short-term effect duration was 3 to 6 months as a typical observation time when receiving VR intervention (25, 27, 30). On average, the total treatment length was 4 weeks.

The AMSTAR-2 analysis (Table 1) was utilized to critically evaluate the methodological quality of included systematic reviews. Reviews were categorized based on the presence of critical and non-critical domains. The analysis revealed 10 articles (17, 19, 20, 22, 23, 28–32) rated with critically low confidence, 5 with low (21, 24–27), and 2 with high (16, 18). None were rated with moderate confidence.

Almost every review demonstrated robust search strategies, which allowed them to consider all relevant studies and avoid potential publication bias. Another strength was the consideration and measurement of the risk of bias in every systematic review, which increased the reliability of their findings. Moreover, each systematic review provided adequate descriptions of the included studies and discussed heterogeneity among the primary articles, thereby creating transparency and enabling recognition of variations. Nevertheless, critical methodological weaknesses were in item 7 (Did the review authors provide a list of excluded studies and justify the exclusions?) and item 15 (If they performed quantitative synthesis did the review authors carry out adequate investigation of publication bias (small study bias) and discuss its likely impact on the results of the review?). This substantially impacted the overall confidence rating. Although the meta-analyses were appropriately conducted, most reviews did not assess publication bias due to the small number of included studies or did not address this aspect. Therefore, the potential impact of selective reporting remains unclear.

The CCA was calculated to assess the overlap of primary studies. A citation matrix is provided in the Supplementary Table 2. The total number of primary studies, including double counting, was 206, and the number of unique studies was 84. The resulting CCA was equal to 9.08%, which indicates a moderate overlap.

**Figure 2:**
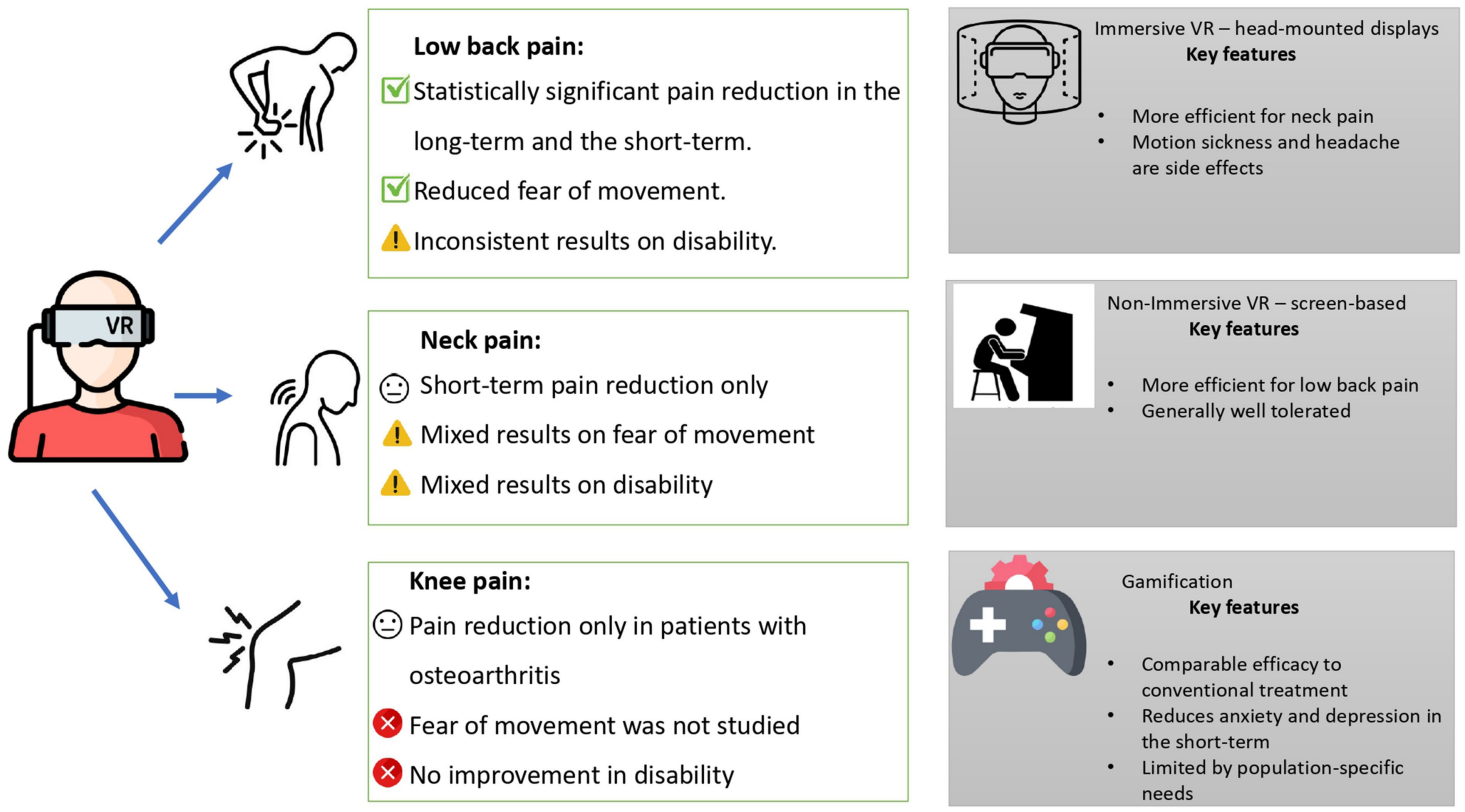
VR efficacy.

The efficacy of VR was summarized based on pain localization, immersion, and gamification in Figure 2. Most of the included systematic reviews and meta-analyses addressed pain in general terms and localization, such as chronic or musculoskeletal neck, back, and knee pain (18–20, 26, 27, 30, 32). However, two studies investigated the ankylosing spondylitis condition, reporting that VR interventions had a positive effect on motor function (16), with immersive VR showing potentially greater benefit (23). Fibromyalgia was addressed in four studies (17, 23, 24, 28). In the first study, gamified VR showed no positive effect in treating fibromyalgia symptoms compared to exercises (17). The second study found immersive VR to be effective in treating fibromyalgia (23), while the third study found that gamified VR was not significant in treating fibromyalgia (24). The fourth study provided moderate evidence to support a positive effect of VR (28). Additionally, two studies focused on inflammation-related pain, specifically in cases of spinal cord injury and herniated discs, and both reported VR to be effective (22, 31). Osteoarthritis was addressed in three studies, two of which showed positive results from VR interventions (25, 29), while one reported no significant effect (21).

### 3.3 VR for low back pain

Bordeleau et al. (16) observed a statistically significant reduction in lower back pain patients compared to controls following moderate immersion VR therapy, with a mean difference (MD) of −0.67 (95% CI: −1.12 to −0.23). Patients reported improved physical performances, better back muscle endurance, and reduced fear of movement (kinesiophobia), which led to a functional capacity to sit, bend, and stabilize the trunk (16). A transient increase in pain during exercises was reported as a single adverse effect. It was proposed that greater therapeutic effects are achieved if more than 12 sessions are conducted. The study has reported high levels of heterogeneity between subgroups (*I*^2^ = 85%) (16). In another study focusing on chronic back pain, VR therapy was reported to be superior to receiving no therapy (Standardized Mean Difference (SMD) at post-intervention = −1.92; 95% CI: −2.73 to −1.11 and SMD at 6 months follow-up = −6.34; 95% CI: −9.12 to −3.56) or oral treatment that included NSAIDs (Nonsteroidal anti-inflammatory drugs) and tramadol (SMD at post-intervention = −0.78; 95% CI: −1.42 to −0.13) (17). No difference has been found between groups that received VR and those with physiotherapy. However, those who received VR and physiotherapy as a combined therapy were shown to have lower pain intensity at the 6-month follow-up session compared to those who received physiotherapy only (SMD = −7.56; 95% CI: −10.79 to −4.32). Patients in all experimental groups using VR post-intervention reportedly experienced a reduced sense of kinesiophobia (MD = −8.96; 95% CI: −17.52 to −0.40). After 6 months, the effect persisted (MD = −12.04; 95% CI: −20.58 to −3.49). However, no significance was found in post-interventional disability (17). The study has also found that specialized simulators, such as the horse-riding simulator (a mechanical device which mimics the horseback riding movement accompanied by VR glasses), yielded better pain reduction compared to interventions without VR. Thus, the study concluded that general VR entertainment devices may be less effective than purpose-built VR devices. The results of Choi et al. (18) align with VR therapy’s pain alleviation ability. Applying VR during the preoperative period has been found to help manage postoperative pain better than conventional methods, reporting reduced emotional discomfort levels after surgery (MD = −1.43; 95% CI: –1.86 to –1.00). Additionally, the study points to implementing VR-based interventions among the younger population, as it has been suggested that adolescents would be more adept with those types of therapies (18).

A different study found the immediate effect of VR-based training on chronic low back pain (MD = −1.43; 95% CI: −1.86 to −1.00; *I*^2^ = 95%), pain-related fear (MD = −5.46; 95% CI: −9.40 to 1.52; *I*^2^ = 90%), and disability (MD -11.50; 95% CI: −20.00 to −3.01; *I*^2^ = 95%) (27). However, it observed that these effects are insignificant in the short term (*p* = 0.16). Such a lack of significance was attributed to an attention bias, as patients returned to their usual activity after completing the first VR session (27). Additionally, one article researched back pain caused by inflammation and found that VR can effectively decrease pain and inflammatory markers levels like C-reactive protein (Weighted mean difference (WMD) = −0.89; 95% CI: −1.07 to −0.7; *I*^2^ = 90%), tumor necrosis factor-alpha (WMD = –6.60; 95% CI: −8.56 to −4.64; *I*^2^ = 0%), and interleukin-6 (WMD = –2.76; 95% CI: −2.98 to −2.53; *I*^2^ = 98%) (31). Yet, changes in fear, disability, and range of motion were found to be insignificant (31). These two studies also indicated high heterogeneity and low-quality evidence as major limitations (27, 31).

### 3.4 VR for neck pain

Hao et al. (30) demonstrated that augmented virtual reality significantly decreases only short-term neck pain (MD = −0.9; 95% CI: −1.31 to −0.58) and improves disability in both the short-term (MD = −2.16; 95% CI: −3.50 to −0.82) and long-term (MD = −2.95; 95% CI: −4.93 to −0.97). However, it was noted that the difference between the control group (standard care) and the VR group was minimal because the control group involved well-established therapeutic exercises. The positive impact of VR was achieved by shifting patients’ focus from movement to their surroundings, enhancing motivation through the novelty and entertainment of tasks, and providing a distraction from pain by immersion (although the intensity of immersion could not be analyzed). The gamified elements of immersive VR were found to promote patients’ active participation and yield better treatment adherence. On the other hand, no significant improvements were found for kinesiophobia, and the generalizability was questionable as the research summarized mixed types of chronic pain. Nine participants (3.7%) reported experiencing “virtual reality sickness” following the interventions that had led to study withdrawal. Strategies such as reducing training session duration while increasing the frequency of interventions were suggested as possible ways of overcoming such issues (30).

Another study analyzed VR-based training on short- and long-term effects in chronic neck pain. In this study, the short-term effect was synonymous with the immediate effect (32). The pooled effect showed that VR-based training, compared to controls, had low-quality evidence for improving disability indexes and was insignificant in decreasing both the immediate and long-term (*p* = 0.10). The kinesiophobia and neck disability index (NDI), however, were found to improve in the long term (SMD for kinesiophobia = −0.19; 95% CI: −0.52 to 0.15, SMD for NDI = −0.49; 95% CI: −1.05 to 0.06). The absence of positive effects for pain management was explained due to sampling differences, with one study including mostly young population with mild disabilities who could adapt quicker to VR headsets and thus rarely develop side effects, while another study involved fighter pilots who had busy schedules, thereby affecting compliance to VR (32).

Ye et al. (20) investigated particularly head-mounted displays in chronic nonspecific neck pain. When comparing preinterventional and postinterventional pain scores, a statistically significant reduction in neck pain (*Z* = 3.46; *p* < 0.001) and improved neck motions with time was observed. Significant improvements in the neck disability index (*Z* = 2.42; *p* = 0.02) and improved flexion/extension (*Z* = 1.96; *p* = 0.05) and rotation (*Z* = 2.43; *p* = 0.02) were reported in groups immediately after receiving VR therapy. However, the results were inconsistent between all included studies, with some reporting no significant difference in control groups that received standard treatment. According to the authors, the extent to which an individual may experience benefits from such therapy varies considerably, challenging accurate estimation of VR-based interventions (20).

The difference in treatment efficiencies between therapy that included VR only and therapy that combined VR with other interventions in neck pain management was investigated (19). The protocols for therapies were based on motivating participants to perform a full range of motion exercises while immersed in different environments, including animal photos or ocean views. When compared to controls that received no therapy, VR therapy favored a statistically significant pain intensity reduction (SMD = −0.51; 95% CI: −0.91 to −0.11). The subgroup analysis showed better pain-decreasing results in patients undergoing VR therapy with other interventions. Lesser disability and higher cervical mean velocity were also reported; however, due to high heterogeneity, no subgroup analysis was performed, suggesting only moderate-quality evidence for the use of VR therapy in MSK disorder management (19).

### 3.5 Low back and neck pain combined

Several studies have analyzed the effects of VR on both low back and neck pain. Henriquez-Jurado et al. (26) concluded that VR-based training effectively reduced low back (SMD = −1.27; 95% CI: −1.45 to −0.8) and neck pain (SMD = −0.45; 95% CI: −0.68 to −0.21) immediately after the first session. However, long-term effects at 1 month (SMD = −1.14; 95% CI: −1.41 to −0.87) and 6 months (SMD = −1.44; 95% CI: −1.7 to −1.18) were observed only in patients with low back pain, but not in patients with neck pain (*p* = 0.41). Interestingly, subgroup analysis revealed that immersive VR is effective only for patients with neck pain (SMD = −0.36; 95% CI: −0.61 to −0.11), but not for patients with low back pain (*p* < 0.01 for both immersive and non-immersive VR). Disability was improved for both types of pain (SMD for back pain = −0.66; 95% CI: −1.26 to −0.1, SMD for neck pain = −0.26; 95% CI: −0.49 to −0.03). Moreover, the effect of VR on lower back pain was increased when combined with alternative therapy. Kinesiophobia and quality of life were improved only for low back pain in the immediate and long term. It was stated that results provide high-quality information (26). Another study by Wong et al. (28) analyzed the effect of VR on chronic pain overall and concluded that VR can decrease chronic pain for both low back pain and neck pain. Immersive VR, when used as an adjuvant, was found to be more effective as a therapy for lower back pain. Wong et al. (28) highlighted that most studies on VR use lacked a mental health condition assessment, provided an insufficient description of VR equipment, and described the long duration of VR sessions, which might have caused discomfort to the patients. The limitations also included a small sample size in some studies, the absence of high-quality research, and the long intervals between VR treatments, as other treatment types used within this timeframe may confound VR long-term outcomes (28).

### 3.6 Knee pain

One study focused on how VR can impact knee pain by Guo et al. (29). The analysis concluded that VR could improve balance (SMD = 0.41; 95% CI: 0.12 to 0.69) and decrease knee pain (SMD = −1.10; CI: −2.02 to −0.18), but cannot improve walking speed (*p* = 0.77) and knee joint range of motion (*p* = 1.00). Notably, the subgroup analysis revealed that patients with osteoarthritis experienced an improvement in knee pain, whereas patients who underwent total knee replacement did not. The study noted that immersion during the training played a significant role in cognitive distraction and could even decrease the heart rate. Overall, the quality of the included articles was high (29).

### 3.7 Immersion

Kantha et al. (23) have focused on active immersive (iVR) and non-immersive VR, which compared to passive, requires active interaction with the virtual environment in MSK disorders. In comparison with no rehabilitation and conventional therapies, overall, pain reduction was observed following iVR application (MD = 9.28; 95% CI: −13.96 to −4.60). In subgroup analysis, more significant pain alleviation was achieved following non-immersive VR than immersive VR (MD = 9.45; 95% CI: −14.57 to −4.33). Non-immersive VR was also shown to reduce psychological distress when compared to no rehabilitation. There was no significant difference between non-immersive and immersive VR in pain outcomes, psychological distress, and functional disability when compared to conventional therapy. Motion sickness and headaches were mentioned as rare side effects. The study reported a level of 93% adherence to the treatment regimen (23).

The study by Lo et al. (25) analyzed the effectiveness of immersive and non-immersive VR, particularly on back, neck, shoulder, hip, and knee pain. The analysis revealed that immersive VR can significantly reduce neck pain (SMD = −0.55; 95% CI: −1.02 to −0.08), whereas non-immersive VR can significantly reduce disability (SMD = −0.44; 95% CI: −0.72 to −0.16), kinesiophobia (SMD = −2.94; 95% CI: −5.20 to −0.68), and pain in low back pain (SMD = −1.79, 95% CI: −2.72 to −0.87). Other regions did not have sufficient studies to draw any conclusion. The biggest drawback was that most chronic low back pain studies used non-immersive VR, while chronic neck pain studies focused on immersive VR – head-mounted displays (25).

### 3.8 Gaming modalities vs. conventional therapies

A study by Mo et al. (21) investigated the effect of commercial and professional rehabilitation exergames on treating MSK pain in the older population (mean age 74.8 ± 6.42). It was found that both the experimental group that underwent exergame sessions and the control group that received conventional treatment reported decreased chronic neck pain, with exergames not being superior to that of exercises (SMD = −0.22; 95% CI: −0.47 to 0.02). No significant difference was found between post-total knee replacement patients who received Nintendo Wii-based exergames and those in a control group that received lower extremities exercises. The authors reported no significant improvements in pain intensity based on the length of training and frequency of sessions in patients of both chronic and non-chronic MSK disorders. It was suggested that more age-related characteristics, such as cognitive and physical decline, should be reflected while designing the exergames, potentially leading to better outcomes if considered (21).

Collado-Mateo et al. (20) have investigated the efficiency of the most popular gaming consoles in pain management of diverse MSK conditions. The exergame protocols have included participants’ active involvement with gaming consoles, such as swinging arms while throwing a bowling ball for patients with upper extremity dysfunction. While most of the included studies have reported pain reduction (SMD = −0.51; 95% CI: −1.25 to 0.23), the high heterogeneity of studies (*I*^2^ = 82%) casts a shadow on concluding the effectiveness of exergames in treating patients with MSK pain (20).

The effect of gamification on the process of treatment was studied in association with pain-related psychological distress (24). Computerized and graphically manipulated image-based interventions verified the short-term impact of such manipulations on reducing anxiety and depression. The study provided very low and low-quality evidence of superiority over other treatments or no treatment in a long-term setting (24).

### 3.9 Mechanisms of VR

**Figure 3:**
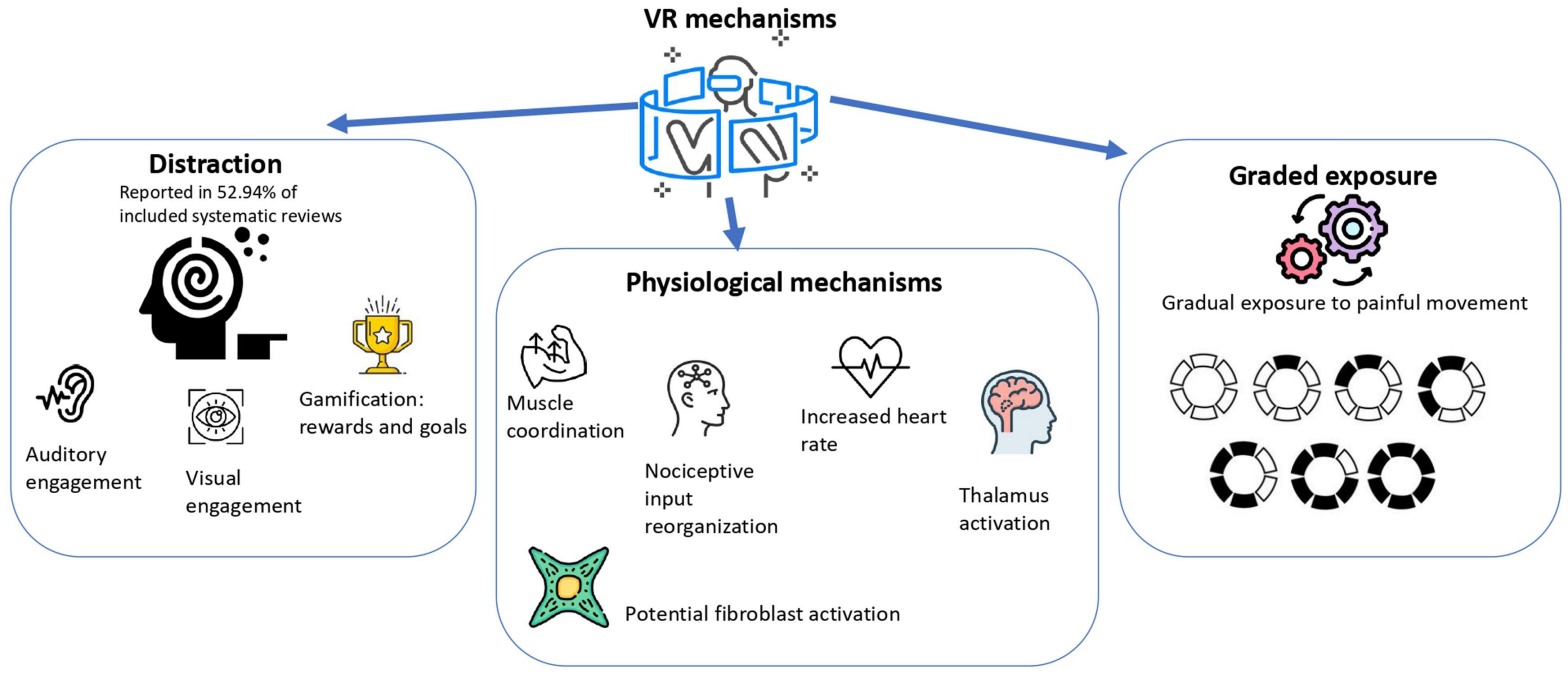
Mechanisms of VR.

The mechanisms of VR to treat MSK pain were reported inconsistently across the included studies. Overall, several hypotheses and observations were proposed, often recurring. The summary of mechanisms is depicted in Figure 3.

The most frequent mechanism was attentional distraction (16–18, 20, 21, 26, 27, 29, 31). VR redirects the patient’s focus from painful stimuli. The distraction was achieved through visual and auditory engagement (31), and concentration on external stimuli (17). Moreover, distraction was enhanced by gamification, like goal-setting and reward-based assignments (16).

Emotional regulation was also described utilizing graded exposure in the setting of chronic pain and fear of movement (21). Patients were gradually exposed to movement that causes pain to make them less sensitive to a stimulus (21).

Additionally, several studies described physiological mechanisms. The mechanisms were: deep and superficial muscles coordination improvement (19), nociceptive input reorganization in sensory and motor brain areas (26). Some studies referred to the regulation of pain via the reduced heart rate and thalamus activation (29). Furthermore, potential activation of fibroblasts through exercise-induced stress adaptation was noted (31).

Most of the studies did not report a specific mechanism (22, 24–26, 30, 32), and overall, VR influence on MSK pain is hypothetical and underexplored.

## 4 Discussion

This umbrella review comprehensively analyzed VR as a treatment for musculoskeletal pain. VR may offer benefits over standard therapies, analgesics, anti-inflammatory drugs, or when compared to placebo in the context of MSK conditions (16–18, 20, 21). Moreover, VR holds a significant promise to improve physical performance. As seen in previous studies, distraction plays a key role (12), but there can also be nondistraction mechanisms involving neurophysiological alterations (33). This is consistent with this review, which implies that such alterations can increase muscle activity or promote myorelaxation. So, the main reason for improvements in MSK patients’ physical activity lies in when VR contributes to muscle functioning, but may also lead to muscle fatigue and overuse, which could result in transient increase in pain – not as a failure of VR, but as a natural adaptation to higher physical load (16). Besides physical benefits, VR is shown to improve mental health. A decrease in fear, emotional discomfort, and kinesiophobia might potentially ameliorate pain catastrophizing since patients will not be afraid to continue exercising. This trend is consistent with previous studies suggesting VR is promising in treating anxiety disorders (8). Although supportive, the mechanisms are not consistently reported across included studies and require further empirical validation. Besides attentional distraction, the included studies proposed other mechanisms, such as graded exposure (21), stress adaptation (31), and neural reorganization (26) mechanisms by which VR could hypothetically alleviate MSK pain. These mechanisms largely align with mechanisms discussed in the previous umbrella review focused on assessing the analgesic effect of VR (34). It confirms attentional distraction as a key mechanism that involves multisensory engagement. It also highlighted neurophysiological effects, such as activation of the insular and sensory cortex and altered nociceptive processing (34).

In terms of MSK conditions and diseases, immersive VR has shown greater potential to improve motor function in patients with ankylosing spondylitis and fibromyalgia, suggesting that depth of engagement may improve therapeutic outcomes (23). However, conflicting results regarding gamified VR, particularly in fibromyalgia, highlight the need for more targeted research to clarify its role (17, 24, 28). Positive effects observed in inflammation-related conditions such as spinal cord injury and herniated discs further support the versatility of VR in pain management. Although results in osteoarthritis have been mixed, overall evidence supports VR as a potentially effective non-pharmacological intervention for a variety of pain conditions.

Depending on localization, VR can manage chronic back pain, back pain after operation, and spinal inflammation, allowing patients to recover their movements (16–18, 26, 27). It seems that VR promotes physical activity as it might use specific equipment like a horse-riding simulator, because it can combine a virtual environment with physical movement that actively engages and promotes mobility in the target area (17). That is why it works better than drugs, but it does not always work better than physical exercise, since usual horse riding can also be a rehabilitation tool. Such observation is consistent with previous results of Tack (2021), who concluded the effectiveness of VR over opioids and equivocal therapeutic mechanisms (35). Non-VR physical exercises may pose an injury risk and result in fear. Since back pain demotivates working out and is usually resistant to painkillers, it is better to use VR as a controlled environment for patients with back pain to decrease the fear of movement when they are assigned for training. Nonetheless, optimized VR interventions for chronic low back pain are suggested to require research with standardized methodology in order to avoid variability in functional improvements and long-term outcomes (16). The same trend is seen when VR is applied to neck pain (19, 20, 26, 30, 32). VR can effectively treat neck pain, but physical exercise seems equally effective (30). Previous study by Galavare et al. (36), where the interdisciplinary rehabilitation program was used, aligns with these results and recommends VR as a support. Therefore, VR can be complementary and does not surpass conventional alternatives. It is advantageous due to its immersive and gamified nature that boosts motivation and adherence through distraction-rich and engaging mechanisms. Nonetheless, the disadvantages involving inconsistency in pain reduction, variability across patient populations, slight impact on kinesiophobia, and side effects like VR sickness decrease the generalizability (19, 20, 30, 32). As part of a multimodal approach, VR demonstrates potential for managing chronic neck pain, where the main strengths are improvements in disability and engagement (19).

Since VR features differential effectiveness for low back and neck pain, the need for personalized approaches emerges (26). VR therapy varies depending on VR modality, pain localization, and integration with physical training. For example, immersive VR may be more relevant to neck pain through engagement of sensory and cognitive mechanisms, whereas both general and immersive VR show positive effects for low back pain if applied consistently (25, 26). Addressing the limitations, like study design improvement, VR protocols standardization, and incorporation of multifaceted mental health evaluation, might increase the reliability of future research outcomes.

Next, it is suggested that VR can be effective for several aspects of knee pain, especially in patients with osteoarthritis (29). This includes pain reduction and improvement in balance, which makes VR intervention a potential tool to alter cognitive and neuromuscular pathways of nociception, proprioception, and motor control. However, the lack of improvement in joint range of motion and walking speed indicates that VR does not change the mechanical or structural aspects of knee functionability. Thus, VR is better used as an additional therapy for a primary intervention like mobility rehabilitation. The aforementioned mechanisms are less relevant for post-surgical knee pain that might involve different neural pathways. Knee pain can also be relieved with distraction and engagement of immersive VR experience (29) which emphasizes psychological components to modulate pain and physiological response. Previous research agrees with these results but points out the limited number of studies conducted on VR as a knee pain treatment to draw reliable conclusions (37).

Besides pain localization, immersive and non-immersive VR were compared in several articles. Generally, non-immersive VR demonstrated better effectiveness in musculoskeletal disorders and in reducing mental health challenges. The reason for this may be because immersive VR requires increased cognitive load and a less tolerable experience for patients, especially during long sessions. Still, VR was found to be more suitable for neck pain. In conditions like neck pain, proprioceptive feedback and engagement play a key role (e.g., looking at objects and turning to follow the moving object). Such varied results have been identified in previous studies to show that VR can be advantageous for a personalized treatment in the specific context, like post-stroke rehabilitation (immersive VR is more suitable) and orthopedic rehabilitation (2D tasks and serious games are more suitable) (38). Nevertheless, discrepancies in the VR types used for specific pain localization (often head-mounted displays for neck pain and rarely for back pain) depict a gap in standardized application protocols. Moreover, there is a lack of sufficient research for other regions, like the shoulder or hip, which limits the generalizability further. To summarize, high adherence and low side effects rates suggest that both immersive and non-immersive VR are well tolerated for any MSK disorder pain management.

Sometimes VR can be defined as gamified and called exergames. Exergames offer pain reduction benefits similar to traditional exercises but do not appear to surpass them in effectiveness (21). This suggests that while exergames can be a viable alternative, they may not provide additional advantages over established rehabilitation methods. Designing age-appropriate exergames could enhance engagement and therapeutic outcomes. The variability in study designs, participant characteristics, and intervention protocols contributes to inconsistent findings regarding the effectiveness of exergames in MSK pain management (22). Previous studies showed that exergames may not be analyzed due to confusion with terminology, but overall show positive results by promoting motivation and engagement (39, 40). Although exergaming is generally safe, enjoyable, and may be appealing to older adults, its effectiveness in reducing musculoskeletal pain remains inconclusive.

### 4.1 Limitations

This umbrella review searched three databases, which might have potentially limited the comprehensiveness of the review. However, selected databases produce a high yield of relevant systematic reviews and extensive overlap with other major databases, which minimizes missing critical studies. Likewise, the search only included English studies, which might have led to language bias. The AMSTAR-2 assessment showed that most of the included reviews were critically low or low quality, primarily due to the lack of critical areas, such as the lack of a list of excluded studies with justification and the lack of evaluation of the publication bias. These methodological disadvantages could introduce selection bias and decrease the overall confidence in the synthesized data. Furthermore, CCA revealed moderate overlap of primary studies which might have introduced redundancy in the evidence base.

### 4.2 Implications

VR generally shows sizeable effectiveness in reducing not only pain, but fear and emotional discomfort, supporting the holistic approach for MSK pain treatment. VR capability for immersion and gamification improves patient engagement and adherence. This offers significant advantages in rehabilitation, namely when motivation is crucial. The choice between immersive and non-immersive VR should involve pain localization and specific rehabilitation goals. For conditions such as chronic neck and back pain, VR is considered the most effective. However, VR should not be considered as a substitute for traditional treatment methods such as physical exercise, but as an additional tool within a multimodal approach. For example, combining VR with mobility rehabilitation or interdisciplinary programs can maximize its benefits.

### 4.3 Future recommendations

Future research should focus on under-researched regions like shoulder or hip pain, and more high-quality studies are needed to clarify VR’s role in treating fibromyalgia and osteoarthritis, where findings remain inconsistent. Likewise, focusing on mental health evaluation will reveal the VR role in alleviating anxiety and depression. The development of universal and comprehensive study designs will ensure consistency (e.g., same duration time and same terminology) and methodological rigor in order to develop optimal VR protocols for MSK pain conditions. Additionally, future studies should standardize the reporting of neurophysiological and psychological mechanisms of VR.

## 5 Conclusion

VR presents a variety of benefits in the treatment of MSK pain, with immersive VR showing promise in conditions such as neck pain, where engagement and distraction are critical. Non-immersive VR may be more effective for low back pain, especially when combined with traditional treatments. However, VR appears less appropriate as a primary intervention for structural or mechanical disorders, like improving joint range of motion in knee pain conditions. Furthermore, future research should pay particular attention to understudied pain sites, methodological consistency, and integration into multimodal treatment plans. For clinical practice, VR may be considered as an additional tool that increases patient motivation and adherence, promoting holistic treatment of musculoskeletal pain.

## Data Availability

The original contributions presented in the study are included in the article/Supplementary material, further inquiries can be directed to the corresponding author.
