# Supplementary material for: Virtual reality for the management of musculoskeletal pain: an umbrella review
Source: Front Med (Lausanne). 2025 Jul 9;12:1572464. doi: 10.3389/fmed.2025.1572464 (PMC12283739; doi:10.3389/fmed.2025.1572464)
Supplement: Supplementary file 2 [file Table_1.docx]

Supplementary Material

**
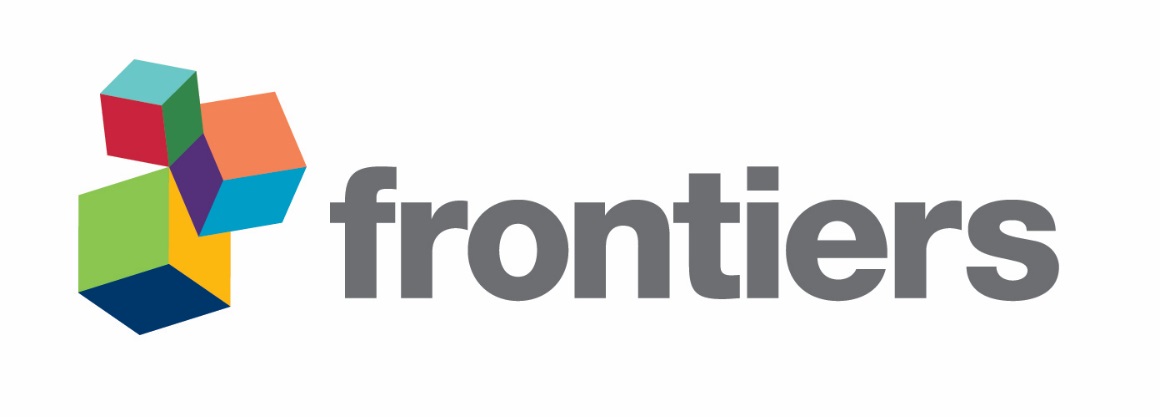
**

**Table 1. Summary of the included studies’ characteristics.**

| **Author, country, year, citation** | **Study design (Systematic review of RCTs or observational studies)** | **Types of musculoskeletal pain** | **Study groups** | **Short description of the protocol** | **N of patients included in the meta-analysis** | **Total number of studies included in the meta-analysis** | **Reported benefits of VR in musculoskeletal pain management** | **Complications (negative effects)** | **Mechanism of VR** | **Study conclusions/comments** |
| --- | --- | --- | --- | --- | --- | --- | --- | --- | --- | --- |
| Ye et al., Canada, 2023 | SR+MA | chronic neck pain | VR vs. control | **Objective**: to determine the effectiveness of VR interventions in improving treatment outcomes for chronic neck pain  Included studies: RCTs, prospective case series  Condition: neck pain  **Population**: adult patients (18-65 years) with neck pain  **Intervention**: VR  **Comparators**: non-exposed group, gold standard physiotherapy and similar treatments  **Outcome**: VAS, NDI, ROM  Quality assessment: Physiotherapy Evidence Database (PEDro) tool and the Downs & Black Quality Assessment Tool  Analysis: treatment effects were measured for continuous variables using mean differences (MD) with 95% confidence intervals (95% CI) | 274, 276, 240, 226 | 3-4 | improved post-intervention VAS scores (Z=3.46, P<0.001; I^2^=2%, χ^2^=3.07, P=0.38); improved NDI scores (Z=2.42, P=0.02; I^2^=92%, χ^2^=26.36, P<0.001); improved ROM for flexion/extension (Z=1.96, P=0.05; I^2^=77%, χ^2^=8.81, P=0.01);  improved ROM for rotation (Z=2.43, P=0.02; I^2^=24%, χ^2^=2.62, P=0.27) | not reported | unclear, hypothetically an attentional distraction | The use of VR interventions has shown improvements in a wide range of outcomes, leading to decreased pain and improved neck flexibility. |
| Mo et al., China, 2023 | SR+MA | chronic pain, back pain, neck pain, musculoskeletal pain, upper extremity dysfunction, post total knee replacement | game technology vs regular activities or traditional physical therapy | **Objective**: to find out if older and middle-aged people can perceive pain better by engaging in exergaming  Included studies: RCTs  Condition: chronic pain  **Population**: 45 years old people without restrictions on pain conditions  **Intervention**: game technology for exercise  **Comparators**: non-technology interventions, usual care, no treatment, and waiting list  **Outcome**: painful effect  Quality assessment: Cochrane Risk of Bias tool in Randomised Trials | 264 | 7 | improvements in chronic neck pain, chronic musculoskeletal pain intensity, thermal pain, chronic low back pain.  Meta-analysis: no statistically significant pain reduction (SMD –0.22; 95% CI –0.47 to 0.02; P=.07) | dizziness, eye pain, or disorientation | unclear, it is likely that exercise results in a rise in stress pain thresholds and leads to a gradual adaptation of central inhibition | Exergaming cannot currently be regarded as an effective technique for reducing pain in older adults due to the paucity of existing data. |
| Guo et al., China, 2023 | SR+MA | neck pain | unimodal (VR therapy), multimodal (VR in combination with other therapies) vs. standard treatment, no intervention | **Objective**: to determine whether VR is effective in treating patients with neck pain  Included studies: RCTs  Condition: neck pain  **Population**: 18 years and older adults with neck pain lasting more than 3 months  **Intervention**: VR  **Comparators**: no treatment, usual treatment, exercise therapy  **Outcome**: pain intensity  Quality assessment: PEDro  Analysis: treatment effects were measured for continuous variables using MD with 95% CI and for dichotomous variables using risk ratios (RR) with 95% CI | 382 | 8 | reduction of pain intensity with multimodal intervention (SMD −0.45, 95% CI −0.78 to −0.13; GRADE: moderate) and clinic or research unit–based treatment (SMD −0.52, 95% CI −0.99 to −0.05; GRADE: moderate);  better analgesic effects among chronic neck pain patients (SMD −0.70, 95% CI −1.08 to −0.32; GRADE: moderate);  less disability (SMD −3.23, 95% CI −4.32 to −2.14; I^2^=46%), lower kinesiophobia (SMD −0.30, 95% CI −0.59 to −0; I^2^=0%), greater cervical range of motion (SMD 0.21, 95% CI 0.08-0.33; I^2^=35%), greater cervical mean velocity (SMD 8.98, 95% CI 2.91-15.06; I^2^=46%), and peak velocity (SMD 10.24, 95% CI 1.28-19.15; I^2^=39%) | motion sickness, simulator sickness and headache | therapeutic effect: greater coordination between the deep and superficial muscles led to a decrease in pain | Moderate-quality data supports the use of VR therapy as a nonpharmacological technique of reducing pain intensity. |
| Choi et al., Korea, 2023 | SR+MA | Low back  pain | VR-based therapy vs. conventional therapy | **Objective**: to determine how VR therapy affects pain relief compared to non-VR therapy  Included studies: RCTs  Condition: pain in medical patients  **Population**: adult patients (> 18 years) with musculoskeletal pain or neuralgia pain  **Intervention**: VR  **Comparators**: surgery, standard therapy, sham control  **Outcome**: pain intensity  Quality assessment: RevMan version 5.4  Statistical considerations: outcomes were measured for continuous variables using MD with 95% CI. The Mantel-Haenszel method was used if the test for heterogeneity was not significant | 1761 | 11 | VR reduces pain intensity ([MD] –1.43; 95% CI –1.86 to –1.00; *I*^2^=95%; *P*<.001), fear (MD –5.46; 95% CI –9.40 to 1.52; *I*^2^=90%; *P*=.007), and disability (MD –11.50; 95% CI –20.00 to –3.01; *I*^2^=95%; *P*=.008) immediately after intervention | motion sickness, nausea, and headache | distraction: reducing pain perception by redirecting attention;  emotional regulation: mitigating negative emotions to reduce postoperative pain and discomfort | VR-based therapy provides moderate to high evidence for pain reduction in comparison to traditional therapies. |
| Kantha et al., Taiwan, 2023 | SR+MA | chronic musculoskeletal disorders | interactive VR (iVR) vs. no rehabilitation or conventional rehabilitation | **Objective**: to investigate the impact of iVR rehabilitation on functional assessments and pain in patients with chronic musculoskeletal conditions  Included studies: RCTs or quasi-RCTs  Condition: musculoskeletal conditions  **Population**: adult patients (>18 years) with musculoskeletal conditions  **Intervention**: iVR  **Comparators**: no treatment or conventional treatment  **Outcome**: pain, functional activities  Quality assessment: PEDro  Analysis: pain outcomes were measured for continuous variables using MD with 95% CI; functional activity and psychological outcomes were calculated using standardized mean difference (SMD) or effect size (ES) with 95% CI | 292, 246, 196, 104, 292, 246 | 2-5 | reduced pain intensity compared to no rehabilitation by an MD of 9.28 (P<0.01, 95% CI=-13.96 to -4.60, Z=3.89, I^2^=0%);  reduced pain intensity compared to conventional rehabilitation by an MD of 8.09 (P<0.01, 95% CI=-12.42 to -3.76, Z=3.66, I^2^=16.3%);  nonimmersive iVR reduced psychological distress compared to no rehabilitation (SMD=-0.35, P=0.04, 95% CI= -0.69 to -0.01, Z=2.03, I^2^=0%) | motion sickness and head-aches (2%) | distraction: diverting patients’ focus from their discomfort | Good quality evidence shows that iVR reduces pain in people with chronic musculoskeletal conditions similar to conventional therapy. |
| Gava et al., Brazil, 2022 | SR+MA | chronic low back pain, chronic neck pain, fibromyalgia, and knee osteoarthritis | digital gaming modalities vs. no treatment or other intervention | **Objective**: to determine whether game-based approaches are effective in improving mental health outcomes in people with musculoskeletal pain  Included studies: RCTs  Condition: video games and psychological wellness  **Population**: adult patients with musculoskeletal pain  **Intervention**: VR  **Comparators**: sham, placebo, other interventions  **Outcome**: pain related fear, catastrophizing, anxiety, depression  Quality assessment: Physiotherapy Evidence Database  Synthesis: GRADE approach | 680 | 13 | reduction in pain-related fear vs. other treatments (SMD: -1.23; 95% CI: -2.02 to -0.44) and anxiety vs. no treatment (SMD: -0.55; 95% CI: -1.01 to - 0.09) | not reported | distraction: redirecting attention from pain to other sensations that reduce cognitive ability to process pain;  graded exposure: stimulating a confrontational response by exposing patients to specific conditions | Interventions using gaming modalities provide low to very low-quality evidence for reducing pain-related fear and anxiety, but not for pain catastrophizing and depression. |
| Brea-Gómez et al., Spain, 2021 | SR+MA | chronic low back pain | VR-based interventions vs. no intervention, interventions without VR, standard treatment, usual care, placebo or control | **Objective**: to investigate the use of gaming therapies in spinal pain patients  Included studies: RCTs  Condition: spinal pain  **Population**: patients with spinal pain  **Intervention**: gaming or VR  **Comparators**: other interventions  **Outcome**: pain, other symptoms, performance  Quality assessment: Downs and Black scale  Statistical considerations: forest chart with heterogeneity assessment | 765 | 11 | reduction in pain intensity at post-intervention (SMD = −1.92; 95% CI = −2.73, −1.11; P < 0.00001) and followup (SDM = −6.34; 95% CI = −9.12, −3.56; P < 0.00001), and lessen kinesiophobia at post-intervention (MD = −8.96; 95% CI = −17.52, −0.40; p = 0.04) and followup (MD = −12.04; 95% CI = −20.58, −3.49; P = 0.006) compared to no VR | nausea, motion sickness, vertigo | distraction: concentrating on an outside stimulation to reduce attention to pain | High heterogeneity findings indicate that after the intervention and at the 6-month follow-up, VR therapies can considerably lessen kinesiophobia and pain severity in individuals with CLBP. |
| Bordeleau et al., Canada, 2022 | SR+MA | back pain | extended reality (XR) vs. comparators | **Objective**: to explore whether mixed and virtual reality are useful rehabilitation techniques for treating back pain  Included studies: quantitative, qualitative, and mixed-method studies  Condition: back pain  **Population**: adult patients with back pain  **Intervention**: Virtual and/or Mixed Reality  **Comparators**: placebo, standard treatment, sham  **Outcome**: movement patterns and intensity of pain  Quality assessment: Cochrane risk of bias tools (ROBINS-I and RoB-2) | 900 | 16 | improvement in the intensity of back pain (Mean Difference: -0.67; 95% CI: -1.12 to -0.23) | a brief, temporary increase in discomfort during exercises | distraction: diverting the brain's attention from harmful signals originating from the body to other sensations;  engaging the working memory: creating the illusion of time speeding up to reduce the length of pain episodes;  illusionization: creating a sense of possessing a virtual body in order to control body perception for pain conditions including changed body image;  gamification: increasing behavioral engagement through achieving in-game goals, objectives and rewards | Studies with high heterogeneity suggest that VR is beneficial for reducing the severity of back pain, and generally has a favorable impact on enhancing other pain outcomes and mobility function. |
| Collado-Mateo et al., Spain, 2018 | SR+MA | chronic pain (low back pain), acute/subacute pain | exergame-based interventions vs. comparators | - | 232 | 6 | reduction in pain (ES: −0.51, 95% CI: −1.25 to 0.23) | not reported | physical activity | There is insufficient evidence that exergame-based exercise reduces musculoskeletal pain due to high heterogeneity in the findings. |
| Lo et al., China, 2024 | SR+MA | Chronic knee, neck, and low back pain | VR assisted active training vs. conventional control | **Objective**: To analyze the effects of immersive and non-immersive VR and overall VR in chronic MSK pain compared to conventional exercises  **Population**: adults with chronic MSK pain  **Intervention**: VR-assisted training  **Comparison**: Active training  **Outcome**: Pain intensity scales, disability and kinesiophobia  Quality assessment: GRADE  Bias: Cochrane RoB | 1144 | 25 | Non-immersive VR reduces pain ([SMD] –1.79, 95% CI –2.72 to –0.87; *P*<.001), improves disability (SMD –0.44, 95% CI –0.72 to –0.16; *P*=.002) and kinesiophobia (SMD –2.94, 95% CI –5.20 to –0.68; *P*=.01) in short term and reduces pain (SMD –8.15, 95% CI –15.29 to –1.01; *P*=.03) and kinesiophobia  (SMD –4.28, 95% CI –8.12 to –0.44; *P*=.03) at 6 months for low back pain  Immersive VR can reduce neck pain (SMD –0.55, 95% CI –1.02 to –0.08; *P*=.02) | Not reported | Not reported | Immersive VR can alleviate neck pain and non-immersive VR can alleviate low back pain but not effective in knee pain. |
| Henríquez-Jurado et al., Spain, 2024 | SR+MA | Chronic low back pain and neck pain | VR based therapy vs. therapeutic exercise, sham and no intervention | **Objective**: to see if the effect of VR is maintained for 1-6 months, find the most appropriate modality and compare VR with alternative methods  **Population**: patients with chronic neck or low back pain  **Intervention**: VR based therapy  **Comparison**: other methos, sham, or no intervention  **Outcome**: pain intensity, disability, kinesiophobia, and QoL  Quality assessment: GRADE and PEDro scales  Bias: PEDro scale | 1522 | 25 | VR reduces pain intensity (SMD = −1.27, 95% CI: −1.45 to −0.8, *P* < 0.001), disability (SMD = −0.66, 95% CI: −1.26 to −0.1, *P* = 0.031), QoL (SMD = 0.62; 95% CI: 0.38 to 0.85; *P* < 0.001), and kinesiophobia (SMD = −0.75, 95% CI: −1 to −0.5, *P* < 0.001) in chronic back pain.  VRBT reduces pain intensity (SMD = −0.45, 95% CI: −0.68 to −0.21, *P* < 0.001), disability (SMD = −0.26, 95% CI: −0.49 to −0.03, *P* = 0.031) in chronic neck pain | Cybersickness | Reorganization of nociceptive input in motor and sensory brain areas, distraction, influence on cognitive pain dimension structures like hippocampus and cortico-limbic system | VR in combination with physical therapy is better than conventional therapy, immersive and non-immersive VR have no difference in effectiveness |
| Li et al., China, 2024 | SR+MA | Chronic low back pain | VR based training with and without physical therapy vs. control | **Objective**: to evaluate short-term effects of VR based training on chronic low back pain  **Population**: patients with low back pain that lasts more than 3 months  **Intervention**: VR based training alone or with physical therapy  **Comparison**: any other methods  **Outcome**: Pain intensity scale, disability, kinesiophobia  Quality assessment: GRADE  Bias: Cochrane RoB | 1059 | 20 | Immediate improvement in chronic low back pain ([MD] –1.43; 95% CI –1.86 to –1.00; *I*^2^=95%; *P*<.001), kinesiophobia (MD –5.46; 95% CI –9.40 to 1.52; *I*^2^=90%; *P*=.007), and disability (MD –11.50; 95% CI –20.00 to -3.01; *I*^2^=95%; *P*=.008) but not in short-term | Not reported | Distraction | VR is effective for chronic low back pain but in the intermediate term |
| Wong et al., China, 2022 | SR | Chronic neck and low back pain, and phantom limb pain | VR vs. comparator | **Objective**: to evaluate effects of VR on chronic pain and mental health  **Population**: adults with chronic pain  Intervention: VR, immersive and non-immersive  **Comparison**: alternative, no intervention, control, or placebo  **Outcome**: pain intensity, anxiety, depression, and mood  Quality assessment: Newcastle-Ottawa scale  Bias: Cochrane collaboration’s tool and Newcastle-Ottawa scale | - (605 in systematic review) | - (17 in systematic review) | Moderate evidence on reducing pain intensity, low evidence on improving depression, anxiety, and mood. | Stated that included studies did not report it | Not reported | VR and particularly immersive VR can reduce pain, but the study designs vary |
| Guo et al., China, 2024 | SR+MA | Knee joint pain | VR or augmented reality vs. standard rehabilitation | **Objective**: to assess the effects of digital healthcare on knee joint pain  **Population**: patients with knee joint pain  **Intervention**: VR or augmented reality  **Comparison**: training  **Outcome**: pain level, ROM, balance during walking  Quality assessment: Cochrane RoB | 473 | 9 | VR improves balance (SMD, 0.41 [0.12, 0.69], p < 0.05) and pain level (SMD, − 1.10 [− 2.02, − 0.18], p < 0.05) | Not reported | Distraction, decline in heart rate.  Regulation of pain input via thalamus | Digital healthcare can significantly improve balance and reduce pain in patients with knee pain and especially in those with osteoporgaosis. Bigger sample size is required. |
| Hao et al., USA, 2024 | SR+MA | Chronic neck pain | VR and conventional training | **Objective**: to compare VR based training and training without VR  **Population**: adults with chronic neck pain  **Intervention**: VR with active training  **Comparison**: physical exercise without VR  **Outcome**: pain intensity, neck kinematics, and patient’s report  Quality assessment: Physiotherapy Evidence Database scale  Bias: Physiotherapy Evidence Database scale | 243 | 6 | VR improves pain intensity in short-term (MD = -0.94, 95% CI: -1.31 to -0.58) and disability in short-term ([MD] = -2.16; 95% confidence interval [CI]n-3.50 to -0.82) and long-term (MD = -2.95; 95% CI: -4.93 to -0.97) | Motion sickness, headache, dizziness | Not reported | VR is significantly better than conventional training for chronic neck pain reduction |
| Zhang et al., China, 2024 | SR+MA | Chronic spinal pain | VR and other conventional therapies | **Objective**: to evaluate VR efficacy for chronic spinal pain and inflammation  **Population**: adults with chronic spinal pain  **Intervention**: VR  **Comparison**: sham and conventional treatment  **Outcome**: pain intensity and inflammatory markers  Quality assessment: Cochrane RoB  Bias: Cochrane RoB | 716 | 15 | VR reduces pain intensity (WMD=–1.63, 95% CI –2.11 to –1.16, P<.001, I2=90%)  C-reactive protein (WMD=–0.89, 95% CI –1.07 to –0.70, P<.001, I2=0%),  Tumor necrosis factor-alpha (WMD=–6.60, 95% CI –8.56 to –4.64, P<.001, I2=98%), and interleukin-6 (WMD=–2.76, 95% CI –2.98 to –2.53, P<.001, I2=0%) | Not reported | Stimulation of sensory system that changes neuroplasticity.  Activation of disc fibroblasts and increase in thickness of multifidus muscle.  Visual and auditory attraction to enhance motor performance | VR therapy is effective in patients with chronic spinal pain caused due to inflammation |
| Opara and Kozinc, Slovenia, 2023 | SR+MA | Chronic neck pain | VR and exercise vs. conventional exercise | **Objective**: to assess VR short- and long-term effects on chronic pain  **Population**: adults with chronic neck pain  **Intervention**: VR with physical exercises  **Comparison**: physical exercise without VR  **Outcome**: pain intensity, ROM, disability, kinesiophobia  Quality assessment: GRADE  Bias: PEDro | 324 | 8 | VR does not reduce pain but moderately improves kinesiophobia (SMD = -0.69, CI = -1.34 to – 0.03) and disability (SMD = -0.95, CI = -1.70 to -0.20) | Not reported | Not reported | VR and conventional exercise have similar effects on chronic neck pain. The quality of included studies is low. |

SR – Systematic review

RCT- Randomised controlled study

VR -Virtual reality

MA – meta-analysis

VAS - Visual Analog Scale

NDI - Neck Disability Index

ROM - Range of motion

MSK – Musculoskeletal pain

RoB – Risk of bias

QoL – Quality of life

SMD – Standardized mean difference

MD – Mean difference

WMD – weighted mean difference

CI – Confidence interval

ES – Effect size

I^2^ - Heterogeneity statistic

P – p-value

CLBP – Chronic low back pain

Z – z-value

χ^2^ – Chi-square test

**Table 2.** Citation matrix

| **Systematic review** | | | | | | | | | | | | | | | | |  |
| --- | --- | --- | --- | --- | --- | --- | --- | --- | --- | --- | --- | --- | --- | --- | --- | --- | --- |
| **Primary Study** | Bordeleau et al., Canada, 2022 | Brea-Gómez et al., Spain, 2021 | Choi et al., Korea, 2023 | Guo et al., China, 2023 | Ye et al., Canada, 2023 | Mo et al., China, 2023 | Collado-Mateo et al., Spain, 2018 | Kantha et al., Taiwan, 2023 | Gava et al., Brazil, 2022 | Lo et al., China, 2024 | Henríquez-Jurado et al., Spain, 2024 | Li et al., China, 2024 | Wong et al., China, 2022 | Guo et al., China, 2024 | Hao et al., USA, 2024 | Zhang et al., China, 2024 | Opara and Kozinc, Slovenia, 2023 |
| Afzal et al. 2022 |  |  |  |  |  |  |  |  |  | 1 | 1 | 1 |  |  |  | 1 |  |
| Alemanno et al. 2019 |  |  | 1 |  |  |  |  |  |  |  |  |  | 1 |  |  |  |  |
| Amin et al. 2017 |  |  |  |  |  |  |  |  |  |  |  |  | 1 |  |  |  |  |
| Battecha et al. 2023 |  |  |  |  |  |  |  |  |  | 1 |  |  |  |  |  |  |  |
| Beltran-Alacreu et al. 2022 |  |  |  |  |  | 1 |  |  |  |  |  |  |  |  |  |  | 1 |
| Birchem et al. 2020 |  |  | 1 |  |  |  |  |  |  |  |  |  |  |  |  |  |  |
| Carvalho et al. 2020 |  |  |  |  |  |  |  |  | 1 |  |  |  |  |  |  |  |  |
| Cikajlo et al. 2016 | 1 |  |  |  |  |  |  |  |  |  |  |  |  |  |  |  |  |
| Chen et al. 2016 |  | 1 |  |  |  |  |  |  |  | 1 |  |  |  |  |  |  |  |
| Collado-Mateo et al. 2017 |  |  |  |  |  |  |  |  | 1 |  |  |  |  |  |  |  |  |
| Cetin et al. 2022 |  |  |  | 1 | 1 |  |  |  |  | 1 | 1 |  |  |  | 1 | 1 | 1 |
| Darnall et al. 2020 |  |  |  |  |  |  |  |  |  |  |  |  | 1 |  |  |  |  |
| Ditchburn et al. 2020 |  |  |  |  |  | 1 |  |  |  | 1 |  |  |  |  |  |  |  |
| Dahl-Popoliozio et al. 2014 |  |  |  |  |  |  | 1 |  |  |  |  |  |  |  |  |  |  |
| Elshazly et al. 2016 |  |  |  |  |  |  |  |  |  | 1 |  |  |  |  |  |  |  |
| Eccleston et al. 2022 |  |  |  |  |  |  |  |  |  |  | 1 | 1 |  |  |  |  |  |
| Fung et al. 2012 |  |  |  |  |  | 1 | 1 |  |  |  |  |  |  |  |  |  |  |
| Garcia-Palacios et al. 2015 |  |  |  |  |  |  |  |  |  |  |  |  | 1 |  |  |  |  |
| Garcia et al. 2021 |  |  | 1 |  |  |  |  |  |  |  | 1 | 1 |  |  |  | 1 |  |
| Garcia et al. 2022 (A) |  |  |  |  |  |  |  |  |  |  | 1 |  |  |  |  |  |  |
| Garcia et al. 2022 (B) |  |  |  |  |  |  |  |  |  |  | 1 |  |  |  |  |  |  |
| Garret et al. 2017 |  |  |  |  |  |  |  |  |  |  |  |  | 1 |  |  |  |  |
| Gianola et al. 2020 |  |  |  |  |  |  |  |  |  |  |  |  |  | 1 |  |  |  |
| Groenveld et al. 2023 |  |  |  |  |  |  |  |  |  |  |  | 1 |  |  |  |  |  |
| Gromala et al. 2015 |  |  |  |  |  |  |  |  |  |  |  |  | 1 |  |  |  |  |
| Gulsen et al. 2020 |  |  |  |  |  |  |  |  | 1 |  |  |  |  |  |  |  |  |
| Hadamus et al. 2021 |  |  |  |  |  |  |  |  |  |  |  |  |  | 1 |  |  |  |
| Hadamus et al. 2022 |  |  |  |  |  |  |  |  |  |  |  |  |  | 1 |  |  |  |
| House et al. 2016 |  |  |  |  |  |  |  |  |  |  |  |  | 1 |  |  |  |  |
| Hsu et al. 2011 |  |  |  |  |  | 1 |  |  |  |  |  |  |  |  |  |  |  |
| Ji-Hyuk et al. 2013 | 1 |  |  |  |  |  |  |  |  |  |  |  |  |  |  |  |  |
| Jordan et al. 2016 |  |  |  |  |  |  |  |  |  |  |  |  | 1 |  |  |  |  |
| Karahan et al. 2016 | 1 |  |  |  |  |  |  | 1 |  |  | 1 |  |  |  |  |  |  |
| Kim et al. 2014 | 1 |  | 1 |  |  |  | 1 |  | 1 | 1 | 1 | 1 |  |  |  |  |  |
| Kim et al. 2020 | 1 | 1 |  |  |  |  |  |  |  | 1 |  | 1 |  |  |  |  |  |
| Li et al. 2021 |  |  |  |  |  |  |  |  |  | 1 | 1 | 1 |  |  |  |  |  |
| Li et al. 2022 |  |  |  |  |  |  |  |  |  |  |  |  |  | 1 |  |  |  |
| Lin et al. 2020 |  |  |  |  |  |  |  |  | 1 | 1 |  |  |  |  |  |  |  |
| Matheve et al. 2020 | 1 |  | 1 |  |  |  |  |  |  | 1 | 1 |  |  |  |  | 1 |  |
| Mbada et al. 2019 | 1 |  |  |  |  |  |  |  |  |  |  |  |  |  |  |  |  |
| Meinke et al. 2022 |  |  |  |  |  |  |  |  |  |  |  | 1 |  |  |  |  |  |
| Mohammad et al. 2018 |  |  |  |  |  |  |  |  |  |  |  |  | 1 |  |  |  |  |
| Monteiro-Junior et al. 2015 | 1 | 1 |  |  |  | 1 | 1 |  |  | 1 | 1 | 1 |  |  |  | 1 |  |
| Mortensen et al. 2015 |  |  |  |  |  |  |  |  |  |  |  |  | 1 |  |  |  |  |
| Mukherjee et al. 2021 |  |  |  | 1 |  |  |  |  |  |  |  |  |  |  |  |  |  |
| Nambi et al. 2020 | 1 | 1 |  |  |  |  |  |  |  | 1 | 1 | 1 |  | 1 |  | 1 |  |
| Nambi et al. 2021 (A) |  | 1 |  |  |  |  |  |  | 1 | 1 |  | 1 |  |  |  |  |  |
| Nambi et al. 2021 (B) |  | 1 | 1 |  |  |  |  |  | 1 | 1 | 1 | 1 |  |  |  | 1 |  |
| Nambi et al. 2021 (C) |  |  |  |  |  |  |  |  |  |  | 1 | 1 |  |  |  | 1 |  |
| Nambi et al. 2022 |  |  |  |  |  |  |  |  |  | 1 |  |  |  |  |  | 1 |  |
| Nusser et al. 2021 |  |  |  | 1 | 1 |  |  |  |  | 1 | 1 |  |  |  | 1 | 1 | 1 |
| Oh et al. 2014 | 1 | 1 |  |  |  |  |  |  |  |  |  | 1 |  |  |  |  |  |
| Ortiz-Catalan et al. 2016 |  |  |  |  |  |  |  |  |  |  |  |  | 1 |  |  |  |  |
| Osumi et al. 2018 |  |  |  |  |  |  |  |  |  |  |  |  | 1 |  |  |  |  |
| Ozlu et al. 2023 |  |  |  |  |  |  |  |  |  |  |  |  |  | 1 |  |  |  |
| Park et al. 2013 |  | 1 | 1 |  |  |  | 1 |  |  | 1 |  | 1 |  |  |  |  |  |
| Park et al. 2020 |  | 1 |  |  |  |  |  |  |  |  | 1 | 1 |  |  |  | 1 |  |
| Pekyavas et al. 2017 |  |  |  |  |  |  |  |  |  | 1 |  |  |  |  |  |  |  |
| Polat et al. 2021 |  |  |  |  |  |  |  |  | 1 |  |  |  |  |  |  |  |  |
| Pournajaf et al. 2020 |  |  |  |  |  |  |  |  |  |  |  |  |  | 1 |  |  |  |
| Punt et al. 2016 |  |  |  |  |  |  | 1 | 1 |  |  |  |  |  |  |  |  |  |
| Raiszadeh et al. 2021 |  |  | 1 |  |  |  |  |  |  |  |  |  |  |  |  |  |  |
| Rezaei et al. 2019 |  |  |  | 1 | 1 |  |  |  |  |  | 1 |  |  |  | 1 |  | 1 |
| Sato et al. 2021 |  | 1 |  |  |  |  |  |  | 1 |  | 1 |  |  |  |  |  |  |
| Sarig Bahat et al. 2015 |  |  |  | 1 |  |  |  |  |  | 1 |  |  |  |  | 1 |  | 1 |
| Sarig Bahat et al. 2018 |  |  |  | 1 |  |  |  | 1 | 1 | 1 | 1 |  | 1 |  | 1 | 1 | 1 |
| Sarig Bahat et al. 2020 |  |  |  | 1 |  |  |  |  |  |  |  |  |  |  |  |  | 1 |
| Shiri et al. 2013 |  |  |  |  |  |  |  |  |  |  |  |  | 1 |  |  |  |  |
| Shim et al. 2023 |  |  |  |  |  |  |  |  |  |  |  |  |  | 1 |  |  |  |
| Soysal Tomruk et al. 2020 |  |  |  |  |  |  |  |  |  | 1 | 1 |  |  |  |  |  |  |
| Stamm et al. 2022 |  |  |  |  |  | 1 |  |  |  | 1 | 1 |  |  |  |  | 1 |  |
| Suh et al. 2018 | 1 |  |  |  |  |  |  |  |  |  |  |  |  |  |  |  |  |
| Thomas et al. 2016 | 1 |  | 1 |  |  |  |  |  |  |  |  |  |  |  |  |  |  |
| Tejera et al. 2020 |  |  |  | 1 | 1 |  |  |  | 1 | 1 | 1 |  |  |  | 1 | 1 | 1 |
| Villiger et al. 2013 |  |  |  |  |  |  |  |  |  |  |  |  | 1 |  |  |  |  |
| Villafaina et al. 2019 |  |  |  |  |  |  |  | 1 |  |  |  |  |  |  |  |  |  |
| Wiederhold et al. 2014 |  |  |  |  |  |  |  |  |  |  |  |  | 1 |  |  |  |  |
| Yalfani et al. 2022 |  |  |  |  |  |  |  |  |  |  | 1 | 1 |  |  |  | 1 |  |
| Yelvar et al. 2017 | 1 |  |  |  |  |  |  |  | 1 |  |  |  | 1 |  |  |  |  |
| Yilmaz et al. 2017 |  |  | 1 |  |  |  |  |  |  | 1 | 1 | 1 |  |  |  |  |  |
| Yoo et al. 2014 | 1 |  |  |  |  |  |  |  |  |  |  | 1 |  |  |  |  |  |
| Yu et al. 2023 |  |  |  |  |  |  |  |  |  |  |  |  |  | 1 |  |  |  |
| Zadro et al. 2019 | 1 | 1 | 1 |  |  | 1 |  | 1 | 1 |  | 1 | 1 |  |  |  |  |  |
| Zavarize et al. 2016 | 1 |  |  |  |  |  |  |  |  |  |  |  |  |  |  |  |  |
